# Neoadjuvant docetaxel, oxaliplatin and S-1 therapy for the patients with large type 3 or type 4 gastric cancer (OGSG1902): protocol of a multi-center, phase II study

**DOI:** 10.1186/s12885-022-09890-w

**Published:** 2022-07-23

**Authors:** Shunji Endo, Tetsuji Terazawa, Masahiro Goto, Ryo Tanaka, Takeshi Kato, Kazumasa Fujitani, Hisato Kawakami, Daisuke Sakai, Yukinori Kurokawa, Toshimasa Tsujinaka, Toshio Shimokawa, Taroh Satoh

**Affiliations:** 1 Department of Digestive Surgery, Kawasaki Medical School, 577 Matsushima, Kurashiki, Okayama, 701-0192 Japan; 2 Cancer Chemotherapy Center, Osaka Medical and Pharmaceutical University, 2-7 Daigaku-machi, Takatsuki, Osaka, Japan; 3 Department of General and Gastroenterological Surgery, Osaka Medical and Pharmaceutical University, 2-7 Daigaku-machi, Takatsuki, Osaka, Japan; 4 Department of Surgery, National Hospital Organization Osaka National Hospital, 2-1-14 Hoenzaka, Chuo-ku, Osaka, Japan; 5 Department of Gastroenterological Surgery, Osaka General Medical Center, 3-1-56 Bandaihigashi, Sumiyoshi-ku, Osaka, Japan; 6 Department of Medical Oncology, Kindai University Faculty of Medicine, 377-2 Ohnohigashi, Osaka-Sayama, Osaka, Japan; 7 Department of Frontier Science for Cancer and Chemotherapy, Osaka University Graduate School of Medicine, 2-2 Yamadaoka, Suita, Osaka, Japan; 8 Department of Gastroenterological Surgery, Osaka University Graduate School of Medicine, 2-2 Yamadaoka, Suita, Osaka, Japan; 9 Department of Surgery, Izumi City General Hospital, 4-5-1 Wake-cho, Izumi, Osaka, Japan; 10 Clinical Study Support Center, Wakayama Medical University, 811-1 Kimiidera, Wakayama, Wakayama Japan

**Keywords:** Stomach Neoplasms, Docetaxel, Oxaliplatin, S-1, Neoadjuvant Therapy, Large type 3, Type 4

## Abstract

**Background:**

Large type 3 and type 4 gastric cancers have extremely poor prognoses. To address this, neoadjuvant chemotherapy may be a promising approach. The phase III JCOG0501 study, conducted to confirm the superiority of neoadjuvant S-1 plus cisplatin followed by D2 gastrectomy over upfront surgery, showed no survival benefit for neoadjuvant S-1 plus cisplatin. In Korea, the PRODIGY study, which was a phase III study of neoadjuvant docetaxel plus oxaliplatin plus S-1 (DOS) followed by surgery and adjuvant S-1 versus surgery and adjuvant S-1 for gastric cancer of T2-3N+ or T4Nany, showed that progression-free survival (PFS) was significantly superior in the neoadjuvant DOS arm. Therefore, DOS therapy may be a promising candidate for preoperative chemotherapy for large type 3 or type 4 gastric cancer.

**Methods:**

Preoperative docetaxel 40 mg/m^2^ and oxaliplatin 100 mg/m^2^ will be intravenously administered on day1 every three weeks. S-1 will be orally administered 80 mg/m^2^ on days 1–14 of a 21-day cycle. Patients will receive three courses of treatment and gastrectomy with ≥D2 lymph node dissection. Postoperative S-1 plus docetaxel therapy (DS) will be administered according to the JACCRO GC-07 (START-2) study. The primary endpoint is the 3-year PFS rate. Secondary endpoints include PFS time, overall survival time, pathological response rate, response rate according to RECIST version1.1, proportion of completion of neoadjuvant chemotherapy, R0 resection rate, proportion of completion of surgery, proportion of completion of protocol treatment, proportion of negative conversion of CY, adverse event occurrence rate, and nutritional evaluation. The null hypothesis for the 3-year PFS rate is 45% and the expected value is 60%. The total sample size is 46 considering that the registration period and follow-up period are two and three years, respectively.

**Discussion:**

This is a prospective, multicenter, single-arm, open-label, phase II trial assessing the efficacy and safety of preoperative DOS and postoperative DS for large type 3 or type 4 gastric cancer. The results will inform future phase III trials and are expected to lead to new treatment strategies for large type 3 or type 4 gastric cancer.

**Trial registration:**

Registered with Japan Registry of Clinical Trials on October 11, 2019 (jRCTs051190060).

## Background

The number of new cases of gastric cancer globally is estimated to be one million per year, and the number of annual deaths is 770,000, which is the third highest among cancer-related deaths [1]. In Japan, the age-standardized mortality rate due to gastric cancer has been declining for both men and women in recent years thanks to screening and eradication of *Helicobacter pylori* [2]. However, the prevention, early diagnosis, and treatment of gastric cancer are still very important.

Type 4 gastric cancer has an extremely poor prognosis. According to a national registry by the Japanese Gastric Cancer Association in 2013, the 5-year survival rate was 23.6% for type 4 gastric cancer, compared with 61.1, 60.9, and 50.6% for types 1, 2, and 3, respectively [3]. Type 3 gastric cancer was reported to show an association between size and recurrence rate [4]. Large type 3 gastric cancer measuring more than 8 cm in diameter has similar biological characteristics to type 4 gastric cancer to develop peritoneal dissemination [5, 6].

The standard treatment for these large type 3 or type 4 gastric cancers is radical gastrectomy and adjuvant chemotherapy, but the outcomes have been unsatisfactory. To improve the poor prognosis of these aggressive types of gastric cancer, neoadjuvant chemotherapy may be a preferable approach in terms of the eradication of micrometastases in addition to local control, higher compliance with intensive chemotherapy, and avoidance of futile surgery by detecting initially invisible distant metastasis after rapid disease progression during neoadjuvant chemotherapy. The Japan Clinical Oncology Group (JCOG) conducted a phase III study, JCOG0501, to confirm the superiority of neoadjuvant S-1 plus cisplatin (SP) followed by D2 gastrectomy over upfront surgery [6]. Although the curative resection rates were 65.1% in the upfront surgery group and 73.5% in the neoadjuvant SP group, the 3-year progression-free survival (PFS) rate and 3-year overall survival (OS) rate, which was the primary endpoint, were 47.7% vs 47.7% (hazard ratio [HR]: 0.976, 95% confidence interval [CI]: 0.738–1.292, *p* = 0.87) and 62.4% vs 60.9% (HR: 0.916, 95% CI: 0.679–1.236, *p* = 0.28), respectively, showing no survival benefit of neoadjuvant SP. Although these results are better than previously reported, they are still unsatisfactory, and further treatment development should improve prognosis.

In Korea, a phase II study of neoadjuvant DOS (docetaxel 50 mg/m^2^ day 1, oxaliplatin 100 mg/m^2^ day 1, and S-1 80 mg/m^2^ day 1–14, every three weeks) chemotherapy followed by surgery and adjuvant S-1 chemotherapy for gastric cancer of cT3–4 N0 or cT2–4 N+ showed that all patients completed three courses of neoadjuvant chemotherapy with an R0 resection rate of 97.6% and pathological complete response of the primary lesion in 19.5% [7]. Thus, DOS seemed a promising neoadjuvant chemotherapy regimen.

Regarding postoperative adjuvant chemotherapy, the JACCRO GC-07 (START-2) study for pStage III gastric cancer showed superiority of docetaxel plus S-1 (DS) therapy to S-1, with 3-year relapse-free survival of 66 and 50%, respectively, at an interim analysis (HR: 0.632, 99.99% CI: 0.400–0.998, *p* ≺ 0.001) [8]. Hence DS therapy as the postoperative treatment seems to be more effective than S-1 monotherapy even for large type 3 or type 4 gastric cancer.

Osaka Gastrointestinal Cancer Chemotherapy Study Group (OGSG) is thus planning a phase II study to confirm the efficacy and safety of preoperative DOS and postoperative DS for large type 3 or type 4 gastric cancer (OGSG1902). The number of patients with large type 3 or type 4 gastric cancer is not large at respective centers, but the treatment outcomes are uniformly not satisfactory. It is forced to accept a study with a low case volume per center. All participating centers are familiar with staging laparoscopy and safe management of pre and postoperative chemotherapy. The results will help in treatment choices for large type 3 or type 4 gastric cancer with poor prognosis.

## Methods/design

**Fig. 1:**
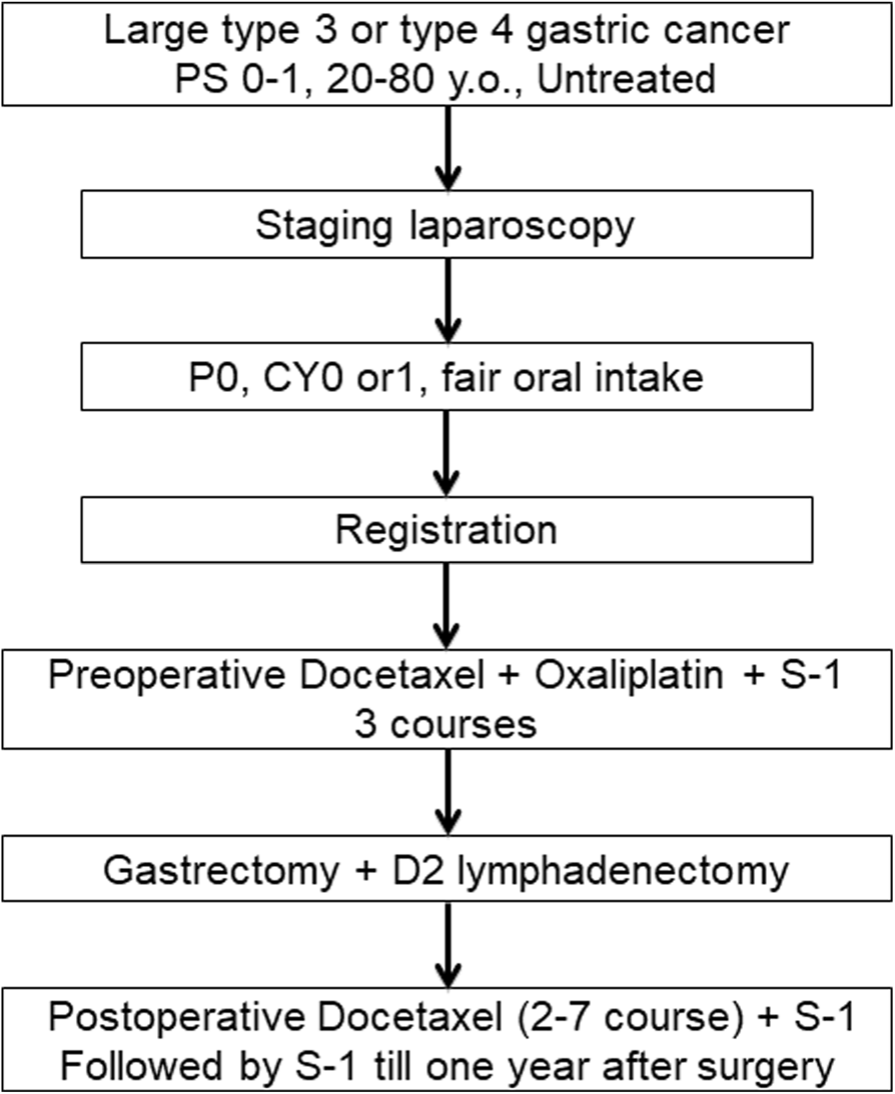
The participant flow diagram. *PS* Eastern Cooperative Oncology Group Performance Status. Clinicopathological findings of gastric cancer are written according the Japanese Classification of Gastric Carcinoma (15th edition) and the surgical procedures are written according to the Japanese Gastric Cancer Treatment Guidelines 2018 (5th edition)

OGSG1902 is a phase II, multicenter, single-arm, open-label, specified clinical trial according to the Japanese Clinical Trials Act, to confirm the efficacy and safety of preoperative DOS and postoperative DS for large type 3 or type 4 gastric cancer. The study design is summarized in Fig. 1. Clinicopathological findings of gastric cancer are documented according to the Japanese Classification of Gastric Carcinoma (JCGC) 15th edition [9]. Surgical procedures are documented according to the Japanese Gastric Cancer Treatment Guidelines 2018 (5th edition) [10]. Tumor response is documented according to Response Evaluation Criteria in Solid Tumors (RECIST) version1.1 [11]. Adverse events are documented according to Common Terminology Criteria for Adverse Events (CTCAE) Version5.0 [12]. Performance status is documented according to Eastern Cooperative Oncology Group (ECOG) [13].

### Endpoints

#### Primary endpoint

The primary endpoint is the 3-year PFS rate. Definition of PFS events is shown in Table 1.

**Table 1:** Definition of PFS event

Preoperative treatment	Surgery	PFS event
non-PD	R0/R1 resection	relapse or death
	R2 resection	relapse, progression, or death
	unresectable/denial of surgery	progression or death
PD	R0/R1 resection	relapse or death
	R2 resection	surgery
	unresectable/denial of surgery	PD judgement

*PFS* progression-free survival, *PD* progressive disease, *R0* no residual tumor, *R1* microscopic residual tumor, *R2* macroscopic residual tumor

#### Secondary endpoints

Secondary endpoints include PFS time (the interval between the date of registration and an event), OS time, pathological response rate evaluated according to JCGC, response rate according to RECIST, completion rate of neoadjuvant chemotherapy, R0 resection rate, completion rate of surgery, completion rate of protocol treatment, negative conversion rate of positive peritoneal lavage cytology, adverse event occurrence rate, and nutritional evaluation.

### Eligibility criteria

The patient inclusion and exclusion criteria are detailed in Table 2.

**Table 2:** Eligibility criteria

Inclusion criteria	
1) Histologically proven gastric cancer (common types)	
2) Large type 3 (≥8 cm measured by CT or endoscopy) or type 4	
3) No peritoneal metastasis (CY0 or 1, and P0) by laparoscopy and CT within 28 days	
4) No sign of distant metastasis including liver metastasis or paraaortic lymph node metastasis	
5) Length of esophageal invasion ≤3 cm by image examination within 28 days	
6) Age between 20 and 80 at registration	
7) Performance status (ECOG) 0 or 1	
8) No prior treatment of chemotherapy or radiation therapy	
9) Adequate organ function (bone marrow, heart, lungs, liver, kidneys, etc.)	
10) Laboratory examination meet the following criteria (data within 14 days from the date of enrollment are used); neutrophils ≥1500 /mm^3^, platelet count ≥100,000 /mm^3^, hemoglobin ≥8.0 g/dL, (Blood transfusion must not be performed within 14 days before the blood sampling date of the test used for registration), AST (GOT) ≤100 IU/L, ALT (GPT) ≤100 IU/L, total bilirubin ≤2.0 mg/dL, and creatinine clearance ≥50 mL/min	
11) Fair oral intake with or without bypass surgery	
12) HER2 negative or not examined	
13) Written informed consent from patient	
Exclusion criteria	
1) Synchronous or metachronous (within 5 years) malignancies	
2) Infectious disease requiring systemic treatment (over 38.0 °C)	
3) Women who are pregnant, may be pregnant, or breastfeeding, or men who want to get pregnant with their partners	
4) Severe mental disease	
5) History of unstable angina pectoris within three weeks or myocardial infarction within six months before registration	
6) Receiving continuous systemic corticosteroid or immunosuppressant treatment	
7) Under treatment with flucytosine, phenytoin, or warfarin	
8) Poorly controlled valve disease, dilated or hypertrophic cardiomyopathy	
9) Hepatitis B surface antigen positive	
10) Interstitial pneumonia, pulmonary fibrosis, or severe emphysema based on chest CT	
11) Poorly controlled hypertension or diabetes	
12) Patients judged inappropriate for the study by the physicians	

*CT* computed tomography, *ECOG* Eastern Cooperative Oncology Group, *AST* Aspartate Aminotransferase, *GOT* Glutamic Oxaloacetic Transaminase, *ALT* Alanine transaminase, *GPT* Glutamic Pyruvic Transaminase, *HER2* human epidermal growth factor receptor 2. Clinicopathological findings of gastric cancer are written according the Japanese Classification of Gastric Carcinoma (15th edition)

### Treatment

#### Preoperative DOS chemotherapy

The first course of preoperative DOS chemotherapy will be started within 14 days after registration. Docetaxel 40 mg/m^2^ for one hour and oxaliplatin 100 mg/m^2^ for two hours will be intravenously administered on day 1 every three weeks. S-1 will be orally administered twice a day at a dose based on body surface area (< 1.25 m^2^, 80 mg; ≥1.25 to < 1.5 m^2^, 100 mg; ≥1.5 m^2^, 120 mg/day) on days 1–14 of a 21-day cycle. Patients will receive three courses of treatment. Aprepitant, serotonin receptor antagonists, long-acting corticosteroids, etc. are recommended to be used as antiemetics. In addition, to prevent hypersensitivity reaction by docetaxel, premedication with long-acting corticosteroids is essential at least for the first administration, and is recommended for the second and subsequent administrations. If the creatinine clearance at the time of registration is ≥50 mL/min and < 60 mL/min, S-1 should be started from one level lower (Table 3).

**Table 3:** Dose reduction level

	Docetaxel	Oxaliplatin	S-1		
Level 0	40 mg/m^2^	100 mg/m^2^	120 mg/body	100 mg/body	80 mg/body
Level − 1	35 mg/m^2^	85 mg/m^2^	100 mg/body	80 mg/body	65 mg/body
Level − 2	30 mg/m^2^	70 mg/m^2^	80 mg/body	65 mg/body	50 mg/body

The second and third courses will be started after confirming that all of the following criteria are met on the day of starting or the day before: body temperature < 38.0 °C, neutrophils ≥1200/mm^3^, platelet count ≥75,000/mm^3^, hemoglobin ≥8.0 g/dL, aspartate aminotransferase (AST) ≤100 IU/L, alanine aminotransferase (ALT) ≤100 IU/L, total bilirubin ≤2.0 mg/dL, creatinine ≤1.5 mg/dL, and CTCAE grade 0–1 fatigue, anorexia, diarrhea, oral mucositis, nausea, vomiting, allergic reaction, pneumonitis, hearing impairment, peripheral motor neuropathy, peripheral sensory neuropathy, and cutaneous toxicity (mottled papular rash, palm / sole redness dyssensitivity syndrome). If any of them is not met, the next course will be postponed until all the criteria are met. If the course cannot be started by day 29, counting from the previous course start date, the doses of docetaxel, oxaliplatin, and S-1 from the next course will be reduced by one level (Table 3). If the course cannot be started by day 43, counting from the previous course start date, the preoperative chemotherapy will be discontinued. Criteria for S-1 pausing, resuming, and skipping, and reduction of docetaxel, oxaliplatin, and S-1 from the next course in the preoperative DOS chemotherapy are shown in Table 4.

**Table 4:** Criteria for S-1 pausing, resuming, and skipping, and reduction of docetaxel, oxaliplatin, and S-1 from the next course in the preoperative DOS chemotherapy

	S-1 pausing	S-1 resuming	S-1 skipping	Docetaxel and S-1 reduction from the next course	Oxaliplatin reduction from the next course
Neutrophil count decreased	Grade 3	Grade 1	–	Grade 4	
Platelet count decreased	Grade 3	Grade 0–1	Grade 4	Grade 3	Grade 2
Creatinine increased	>1.5 mg/dL	≦1.5 mg/dL	>2.0 mg/dL	>1.5 mg/dL	
Febrile neutropenia	–	–	Grade 3	Grade 3	
Infection	–	–	Grade 3	Grade 3	
Diarrhea, Oral mucositis, Rash	Grade 2	Grade 0–1	Grade 3	Grade 3	
Nausea, Fatigue	Grade 3	Grade 0–1	–	Grade 3	
Vomiting, Anorexia	Grade 3	Grade 0–1	–	Grade 3	
Infusion reaction	Grade 2	Grade 0–1	Grade 3	–	
Peripheral motor neuropathy	–	–	–	–	Grade 2,3
Peripheral sensory neuropathy	–	–	–	–	Grade 2,3
Hypernatremia, Hyponatremia, Hyperkalemia, Hypokalemia	Grade 3	Grade 0–1	Grade 4	Grade 3	

*DOS* docetaxel, oxaliplatin, and S-1. Grades are written according to Common Terminology Criteria for Adverse Events (CTCAE) Version5.0

#### Surgery

After confirming that R0 resection is possible by image evaluation after the final preoperative chemotherapy, gastrectomy with ≥D2 lymph node dissection will be performed within 56 days (recommended within 28 days) from the last administration of S-1 in the final course. If R0 resection is impossible or if distant metastases including peritoneal metastases (P1), hepatic metastases (H1) and positive peritoneal cytology (CY1) are found during surgery, the protocol treatment will be discontinued.

#### Postoperative DS chemotherapy

Postoperative DS chemotherapy will be started within 42 days after surgery. The regimen is based on the START-2 study [8]. Docetaxel 40 mg/m^2^ for one hour will be intravenously administered on day 1 every three weeks, starting from the second course. S-1 will be orally administered twice a day at a dose based on body surface area (< 1.25 m^2^, 80 mg; ≥1.25 to < 1.5 m^2^, 100 mg; ≥1.5 m^2^, 120 mg/day) on days 1–14 of a 21-day cycle and started from the first course. If the creatinine clearance is ≥50 mL/min and < 60 mL/min, the dose of S-1 will be reduced by one level (Table 3). From the eighth course, S-1 alone will be continued on day 1–28 of a 42-day cycle until one year after surgery.

The first course of postoperative chemotherapy will be started after confirming that all of the following criteria are met; CTCAE grade 0–1 anorexia, ECOG PS 0–1, body temperature < 38.0 °C, neutrophils ≥1500 /mm^3^, platelet count ≥75,000 /mm^3^, hemoglobin ≥8.0 g/dL, AST ≤100 IU/L, ALT ≤100 IU/L, total bilirubin ≤2.0 mg/dL, and creatinine clearance ≥50 mL/min. If S-1 cannot be started within 42 days after surgery due to surgical complications or delayed histopathological diagnosis of the resected specimen, the protocol treatment will be allowed to be postponed until day 84. If S-1 cannot be started by day 84, the protocol treatment will be discontinued.

The second or later courses will be started after confirming that all of the following criteria are met on the day of starting the course or the day before: body temperature < 38.0 °C, neutrophils ≥1000/mm^3^, platelet count ≥75,000/mm^3^, hemoglobin ≥8.0 g/dL, AST ≤100 IU/L, ALT ≤100 IU/L, total bilirubin ≤2.0 mg/dL, creatinine ≤1.5 mg/dL, and CTCAE grade 0–1 diarrhea, nausea, vomiting, anorexia, oral mucositis, and other non-hematological toxicity. If any of these criteria are not satisfied, the treatment will be postponed. If the next course cannot be started within 28 days, counting from the planned starting date, the protocol treatment will be discontinued. Criteria for S-1 skipping and reduction of docetaxel and S-1 from the next course in the postoperative DS chemotherapy are shown in Table 5. After 8 courses of DS chemotherapy, S-1 monotherapy with ‘4 weeks administration and 2 weeks off’ schedule will be started. Criteria for skipping and reduction are the same as in Table 5 and ‘4 weeks administration and 2 weeks off’ schedule can be modified to ‘2 weeks administration and 1 week off’ schedule.

**Table 5:** Criteria for S-1 skipping and reduction of docetaxel and S-1 from the next course in the postoperative DS chemotherapy

	S-1 skipping	Docetaxel and S-1 reduction from the next course
Neutrophil count decreased	Grade 3	Grade 4
Platelet count decreased	Grade 3	Grade 3
Creatinine increased	>1.5 mg/dL	>1.5 mg/dL
Febrile neutropenia	–	Grade 3
Infection	–	Grade 3
Diarrhea, Oral mucositis, Rash	Grade 2	Grade 3
Nausea, Fatigue	Grade 2	Grade 3
Vomiting, Anorexia	Grade 2	Grade 3
Infusion reaction	Grade 2	–
Hypernatremia, Hyponatremia, Hyperkalemia, Hypokalemia	Grade 2	Grade 3

*DS* docetaxel and S-1. Grades are written according to Common Terminology Criteria for Adverse Events (CTCAE) Version5.0

### Follow-up

Patients will be followed up on a fixed schedule for three years after accrual completion. Physical and blood examinations will be done every three months. An enhanced chest and abdominal CT will be done every six months.

### Study design and statistical considerations

The null hypothesis is that “the 3-year PFS rate with this protocol treatment is 45%”, since the 3-year PFS rate of the neoadjuvant group in JCOG0501 was 47.7%. The expected value is set to 60% because three courses of toxic preoperative DOS therapy have been added, and DS therapy showed a 16% increase of 3-year relapse-free survival over S-1 in the START-2 study [8]. With α = 0.10, 1-β = 0.8, registration period of two years, and follow-up period of three years, the minimum sample size is taken to be 44. The total sample size is set to 46 to account for deviation.

The one-sided alternative hypothesis that “3-year progression-free survival with this protocol treatment exceeds 60%” will be evaluated by one-sample log-rank test with a significance level of 0.10. The *p*-value (null distribution) will be calculated by exact tests. If the null hypothesis is rejected, the treatment will be judged to be valid, and if it is not rejected, it will be judged to be invalid.

All statistical analyses will be conducted at the OGSG Data Center.

### Monitoring

The Data and Safety Monitoring Committee of the OGSG will independently review protocol compliance, safety of the study, and the accuracy of data collection. This monitoring will be performed annually.

## Discussion

OGSG1902 is the first phase II study to investigate the efficacy and safety of preoperative DOS and postoperative DS for large type 3 or type 4 gastric cancer.

In the JCOG0501 study [6], two courses of neoadjuvant SP treatment did not show superiority to upfront surgery treatment for large type 3 or type 4 gastric cancer. The reasons were that SP therapy with a four-week cycle may not have sufficient treatment intensity to control micrometastasis and the duration of combination therapy was short. The treatment results may be improved by strengthening preoperative and postoperative chemotherapy. Triple therapy is expected to improve the histological response compared with dual therapy, and will be an important treatment strategy for gastric cancer treatment. The standard treatment for gastric cancer in Europe is docetaxel, oxaliplatin, fluorouracil, and leucovorin therapy (FLOT), with pathological complete regression observed in 16% [14]. And in East Asia, DOS is considered as a promising triple therapy.

After starting OGSG1902, the PRODIGY study, a phase III trial of neoadjuvant DOS plus surgery and adjuvant S-1 versus surgery and adjuvant S-1 for gastric cancer of T2-3N+ or T4Nany, showed that PFS was significantly superior in the neoadjuvant DOS arm (HR for PFS adjusted for stratification factors: 0.70, 95% CI: 0.52–0.95, stratified log-rank *p* = .023) [15]. Three-year PFS rates were 66.3% with neoadjuvant DOS and 60.2% with upfront surgery.

In Japan, the JCOG1704 study, a phase II trial to evaluate the efficacy of preoperative DOS for gastric cancer with resectable extensive lymph node metastases, is ongoing [16]. As febrile neutropenia occurred in 9.8% of patients in the Korean phase II study with docetaxel 50 mg/m^2^ [7], they reduced the docetaxel dose to 40 mg/m^2^ in the JCOG1704 study. Considering that the postoperative docetaxel dose in the START-2 study was 40 mg/m^2^ [8], we also adopted docetaxel 40 mg/m^2^ in our preoperative DOS regimen.

OS is considered the most reliable endpoint. However, the disadvantage of using OS as the endpoint is that it requires an extended follow-up period. Disease-free survival was reported to be an acceptable surrogate for OS in trials of adjuvant chemotherapy for gastric cancer [17]. In clinical trials of preoperative treatment, however, not all cases become cancer-free and PFS is more common than disease-free survival as an endpoint. Furthermore, most progression events occurred within three years (3-year PFS rate 47.7%) in JCOG0501 [6]. Based on the above, the 3-year PFS rate was taken as the primary endpoint of this study.

This study includes patients with CY1, considering that JCOG0501 also included CY1 cases and that a better prognosis can be expected if surgery is performed when CY1 is converted to CY0 by chemotherapy. We also plan to analyze whether there is a difference in prognosis between CY1 and CY0. P1 is not included in this study because it is difficult to eliminate peritoneal dissemination by chemotherapy. Patients with obstruction requiring for bypass surgery may have more advanced cancer and poorer nutritional or general condition than those without obstruction. Since the purpose of this study is to explore the optimal treatment using an oral agent (S-1) for patients with aggressive disease, we considered that those who have underwent bypass surgery should be qualified. Bypass surgery might be a confounding factor, and therefore it may be interesting to test its possibility if an appropriate number of cases were enrolled.

The OGSG1902 study will provide new information on the efficacy and safety of preoperative DOS and postoperative DS for large type 3 or type 4 gastric cancer. A future randomized phase III study will be planned based on the results.

## Data Availability

The datasets used and/or analyzed during the current study are available from the corresponding author upon reasonable request.

## Abbreviations

JCOG: Japan Clinical Oncology Group
SP: S-1 plus cisplatin
PFS: progression-free survival
OS: overall survival
HR: hazard ratio
CI: confidence interval
DOS: docetaxel, oxaliplatin and S-1
DS: docetaxel and S-1
OGSG: Osaka Gastrointestinal Cancer Chemotherapy Study Group
JCGC: Japanese Classification of Gastric Carcinoma
RECIST: Response Evaluation Criteria in Solid Tumors
CTCAE: Common Terminology Criteria for Adverse Events
ECOG PS: Eastern Cooperative Oncology Group Performance Status
AST: aspartate aminotransferase
ALT: alanine aminotransferase
